# Supplementary material for: Structural basis of α-latrotoxin transition to a cation-selective pore
Source: Nat Commun. 2024 Oct 3;15:8551. doi: 10.1038/s41467-024-52635-5 (PMC11449929; doi:10.1038/s41467-024-52635-5)
Supplement: Supplementary file 1 — Supplementary Information [file 41467_2024_52635_MOESM1_ESM.pdf]

Supplementary Information

of

Structural basis of α-latrotoxin transition to a cation selective pore

BU Klink, A Alavizargar, KS Kalyankumar, M Chen, A Heuer and C Gatsogiannis

Contents:

1. Supplementary Figures

2. Supplementary Tables

3. Supplementary References

1. Supplementary Figures

Supplementary figure 1

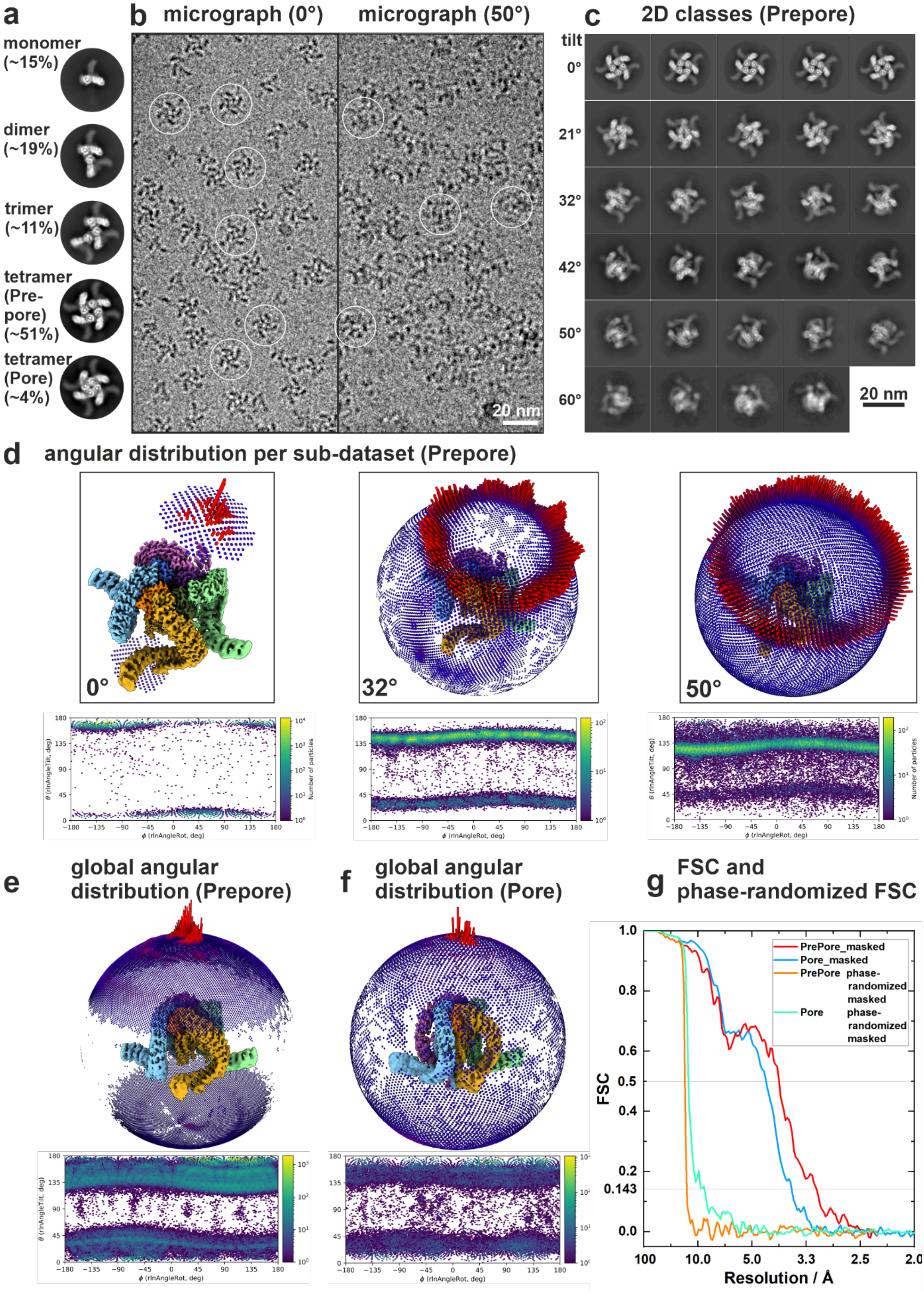

Supplementary figure 1: Cryo-EM analysis of α-LTX. a Representative reference-free 2D class averages and distribution of the different oligomeric states from the 0° dataset. Note that the 2D class averages of tetramers show exclusively top-views. **b** Representative cryo-EM micrographs of α-LTX from the untilted and 50° tilted dataset. **c** 2D class averages from datasets collected at 0°, 21°, 32°, 42°, 50°, 60° **d** Distribution of particles in 3D and 2D histogram representation for the 0°, 32° and 50° datasets. **e,f** Particle distributions for the final reconstructions of the prepore and pore state from the merged dataset. **g** masked and phase-randomized masked half-map FSC curves (calculated by Relion Postprocess) of the final reconstructions.

Supplementary figure 2

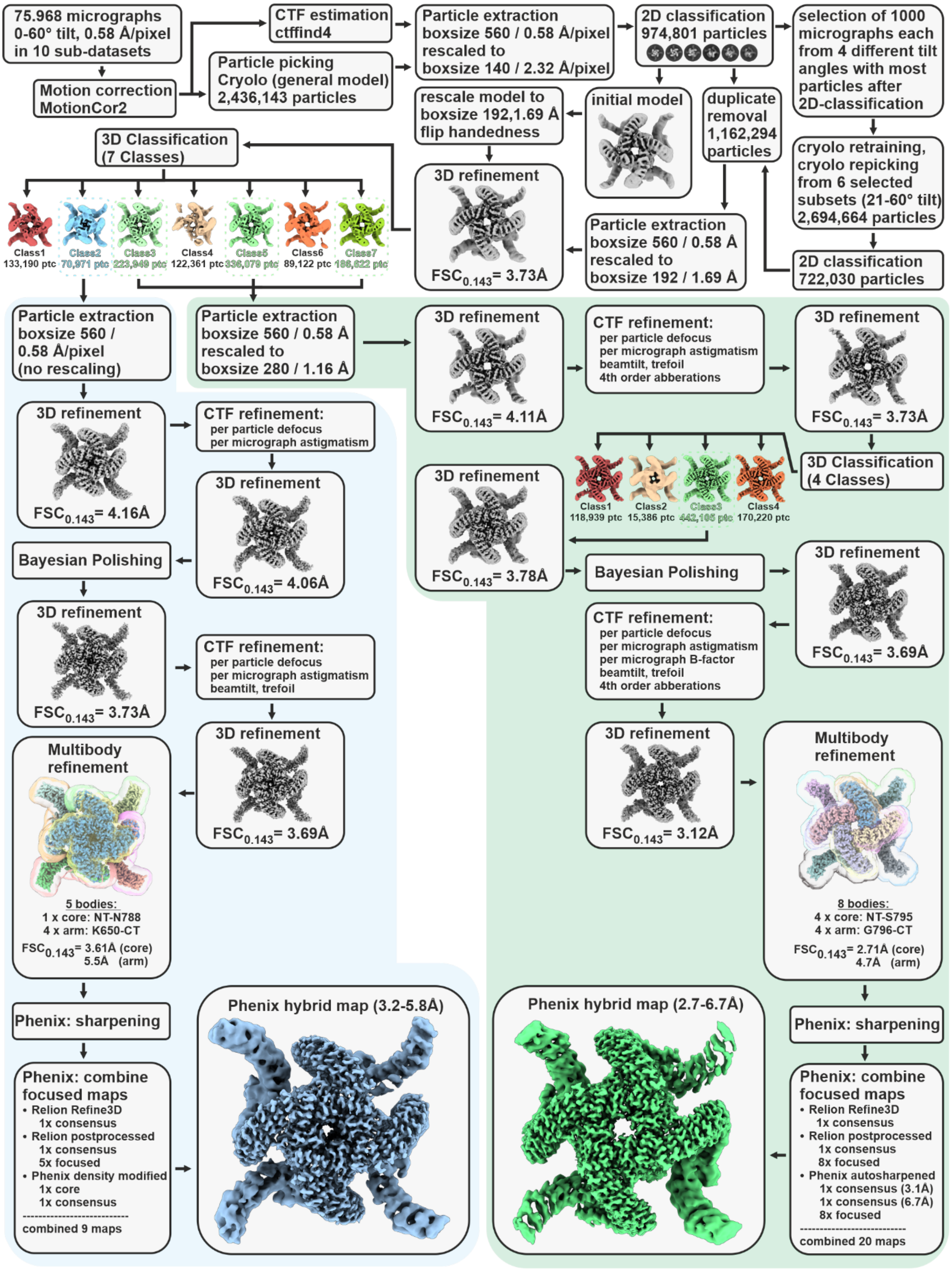

Supplementary figure 2: Cryo-EM processing workflow for structure determination of **α-LTX in prepore and pore state.** The flowcharts of α-LTX prepore and pore processing workflows are highlighted with a green and blue background, respectively.

Supplementary figure 3

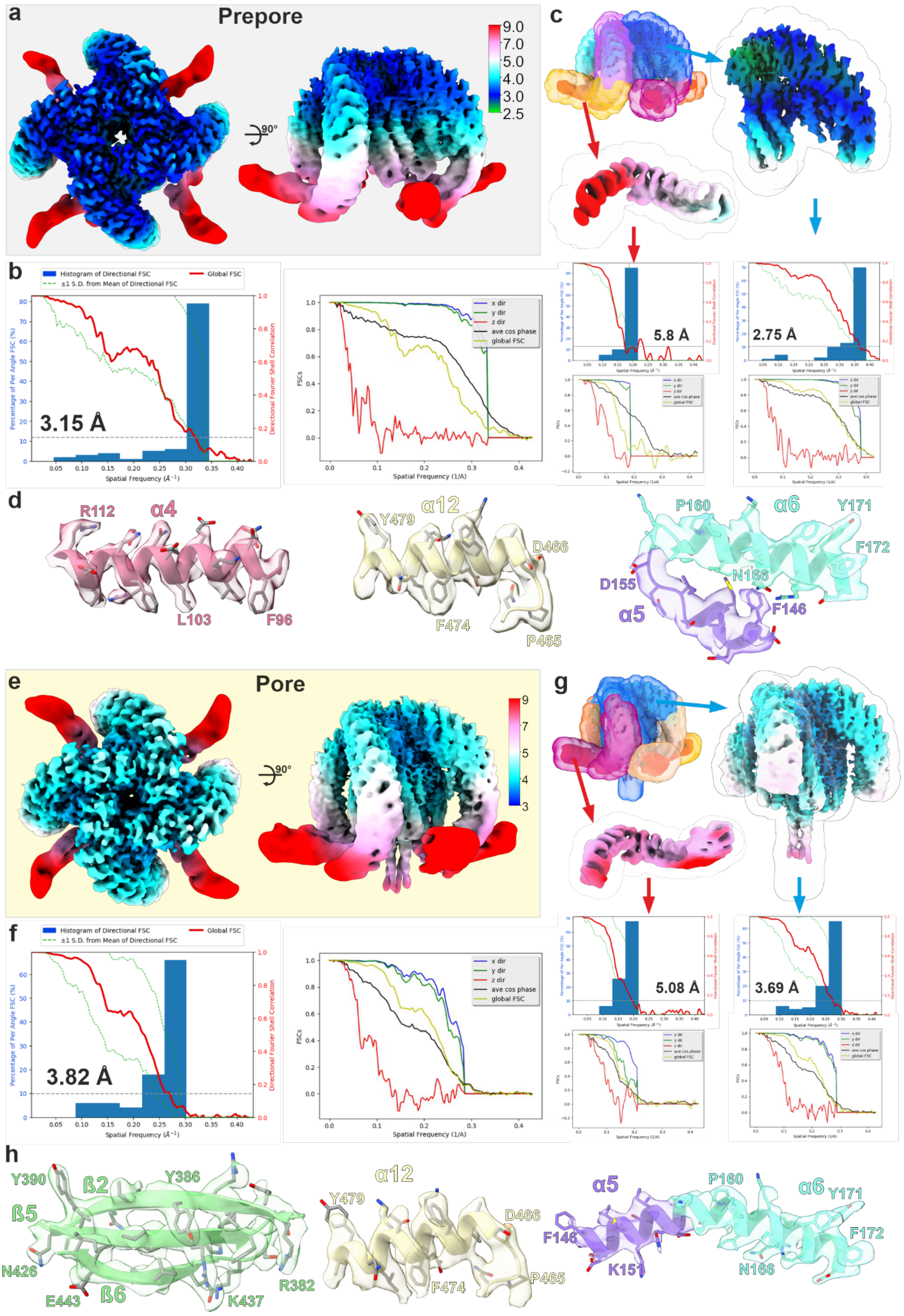

Supplementary figure 3: Resolution of cryo-EM maps. a,b Density map of α-LTX prepore prior multi-body refinement colored by local resolution (**a**) and respective 3D-FSC[1] (**b**). **c** 3D masks used for multi-body refinement of the prepore state in RELION4 and the resulting locally refined bodies colored by local resolution, together with their 3D-FSCs. **d** Superposition of segments of the molecular model of the prepore with the composite map for representative regions of the structure with varying local resolution. **e-f** Density map of α-LTX pore prior multi-body refinement colored by local resolution (**e**) and respective 3D-FSC (**f**). **g** 3D masks used for multi-body refinement of the pore state in RELION4 and the resulting locally refined bodies colored by local resolution, together with their 3D-FSCs. **h** Superposition of segments of the molecular model of the pore with the composite map for representative regions of the structure with varying local resolution.

Supplementary figure 4

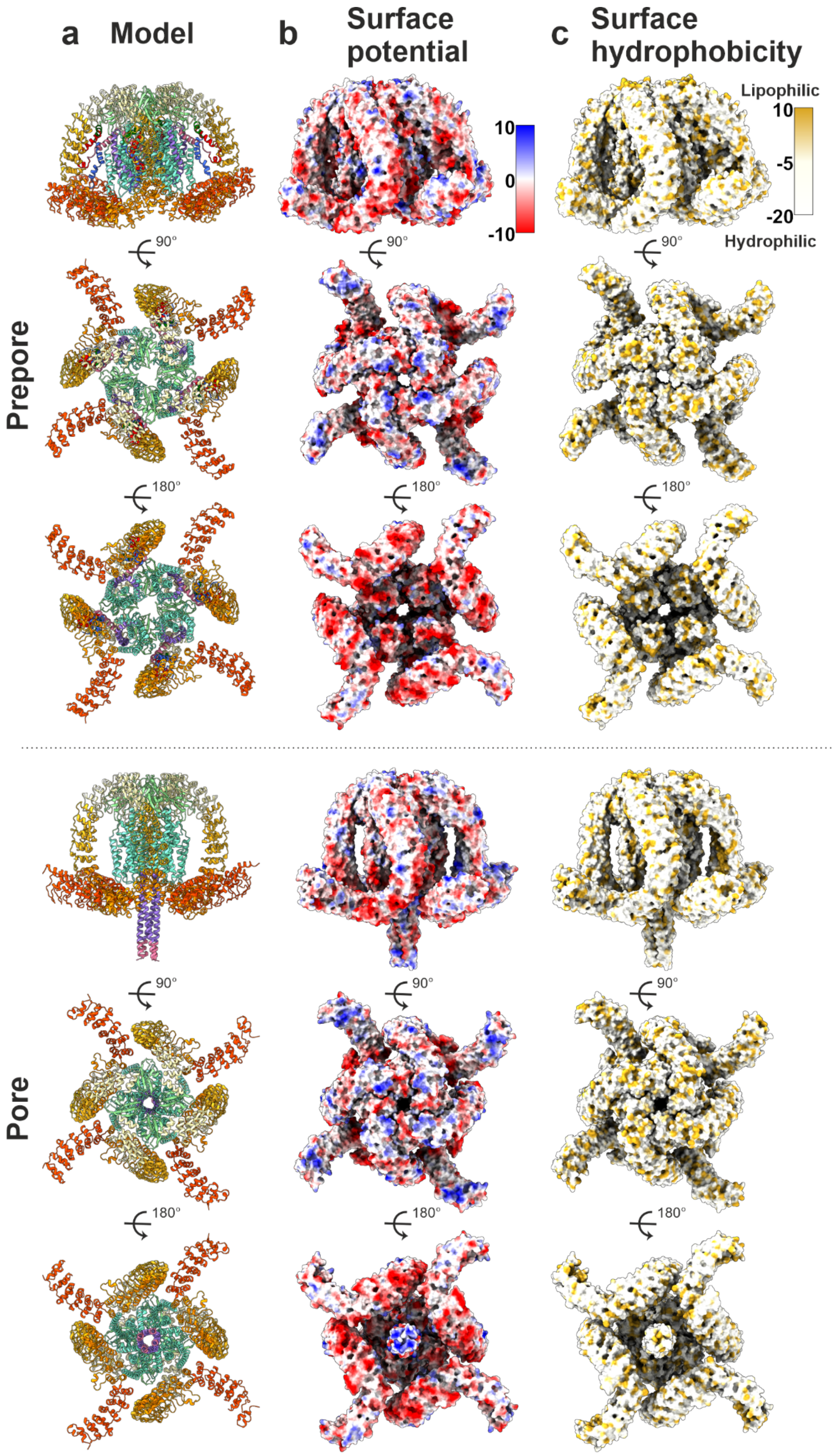

Supplementary figure 4: Biophysical properties of α-LTX prepore and pore state. a-c Molecular model (**a**), surface coulombic electrostatic potential at 298K [kcal/(mol·*e*)] (**b**) and Molecular Lipophilicity Potential (MLP) maps (**c**) of the cryo-EM structures in prepore and pore state.

Supplementary figure 5

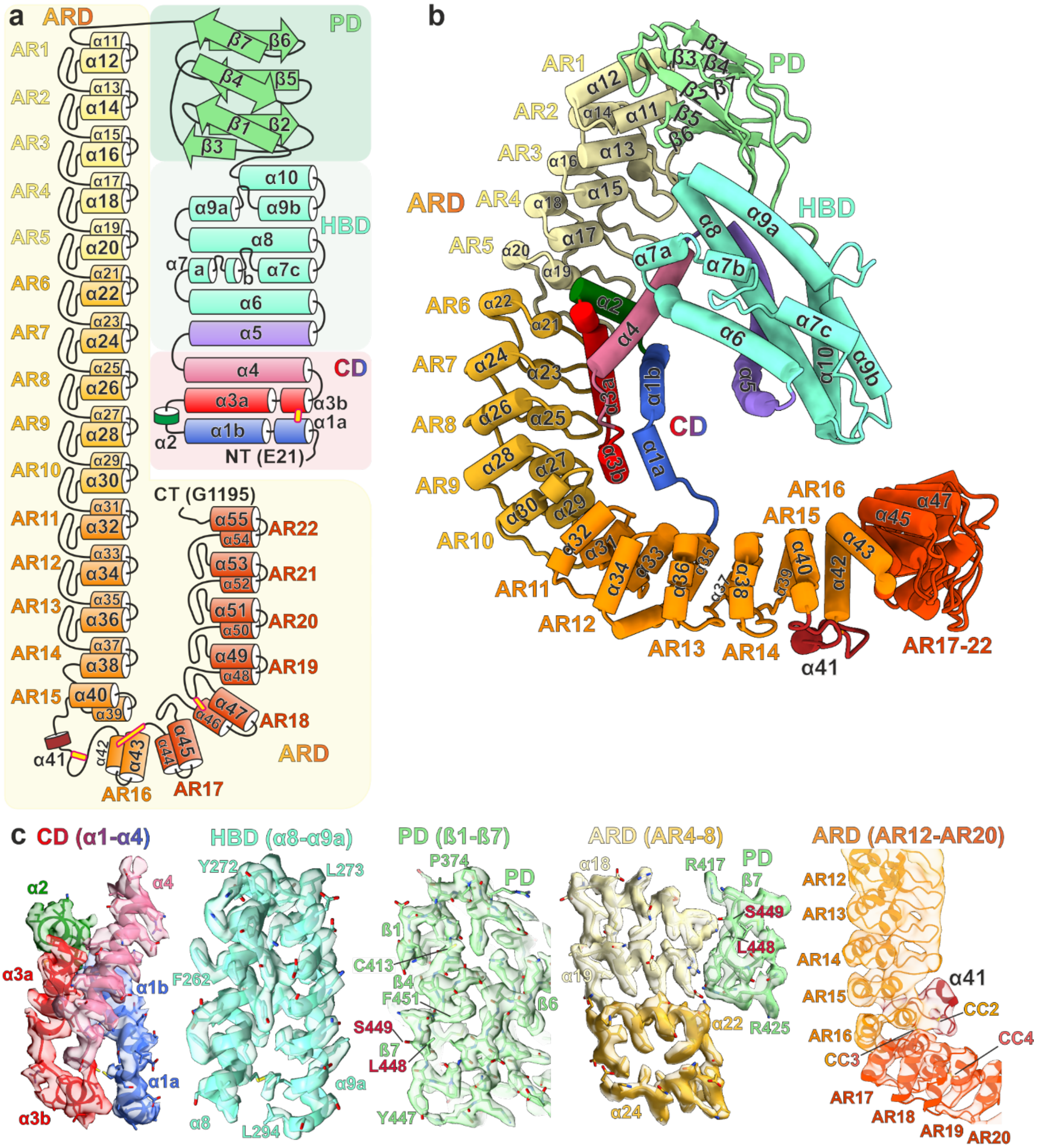

Supplementary figure 5: Architecture of one subunit of α-LTX tetramer in the prepore state. a,b Topology diagram **(a)** and ribbon representation (**b**) of α-LTX in the prepore state. Connector domain (CD), helical bundle domain (HBD; cyan), plug domain (PD; green) and ankyrin-like repeat domain (ARD; brown spectrum). The nomenclature and color code is used throughout the manuscript. **c** Superposition of segments of the α-LTX prepore cryo-EM density map with the corresponding molecular model for the different domains of the subunit.

Supplementary figure 6

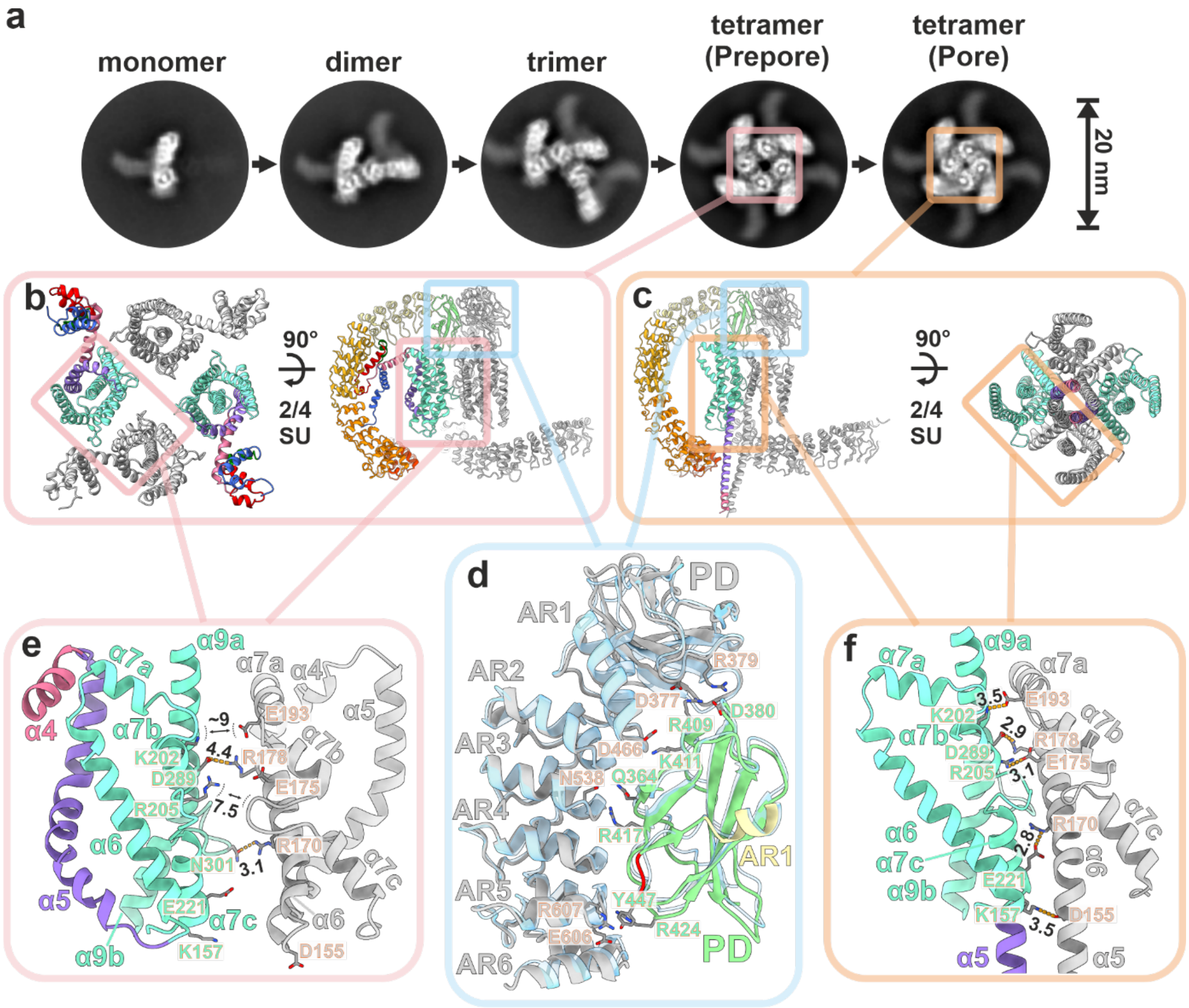

Supplementary figure 6: Assembly and stabilization of tetrameric α-LTX in the prepore and pore state. a Stepwise assembly of α-LTX prepore and transition to the pore state. Representative reference-free 2D class averages are shown. **b,c** Top view of the central core of the tetrameric complex (HBD and CD domains) and side view of two clockwise neighboring monomers in α-LTX prepore (**b**) and pore (**c**) state. **d** Interactions between monomers are dominated by contacts between the PD of one subunit with the ARD of the clockwise neighboring monomer. This interface remains stable during the prepore(colored)→pore(grey) transition. **e-f.** Interactions between the HBD domains of adjacent monomers in the prepore (e) and pore (f) state. HBD interactions are rather weak in the prepore state (**e**), but in the pore state neighboring HBD domains move closer together to form a tight interface (**f**).

Supplementary figure 7

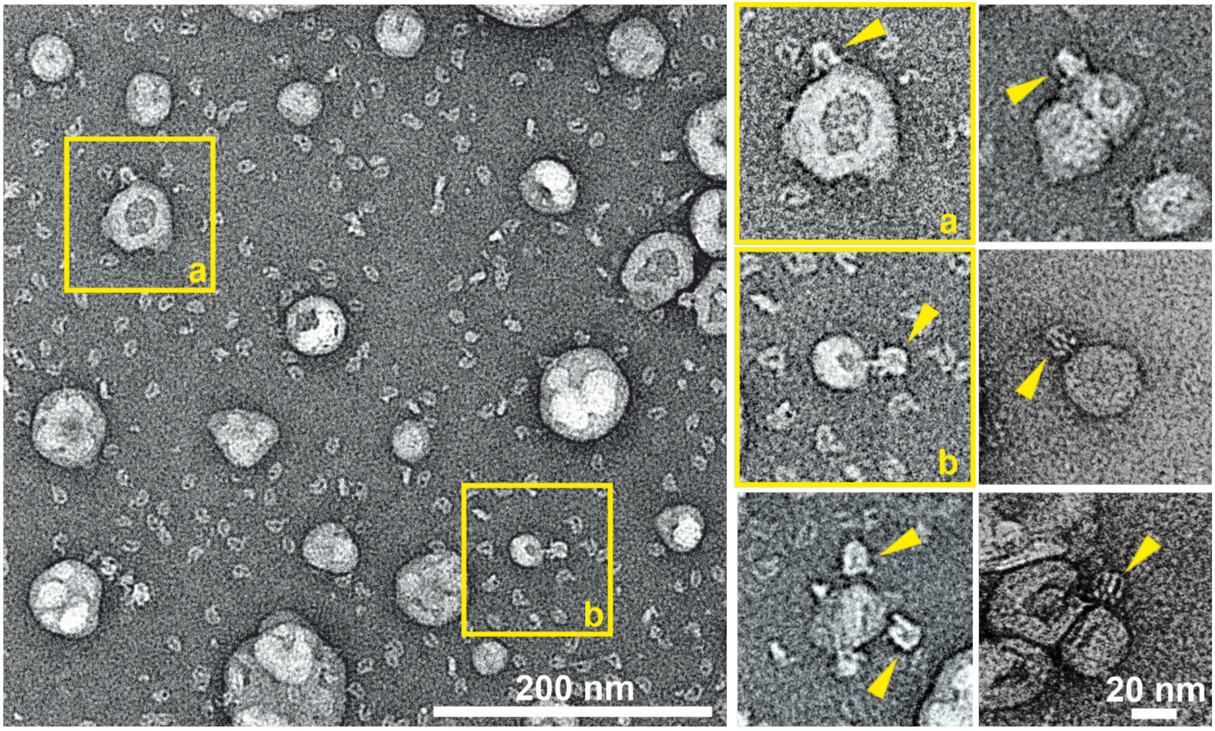

Supplementary figure 7: α-LTX reconstituted in liposomes. Representative negative stain EM micrograph (left) and images of individual liposomes (right) with incorporated α-LTX particles (yellow arrows). Particles interacting with the membrane show the characteristic umbrella-like shape of the tetrameric pore. The vast majority of particles are distributed in the background and show the characteristic G-shape of the monomer in the prepore state.

Supplementary figure 8

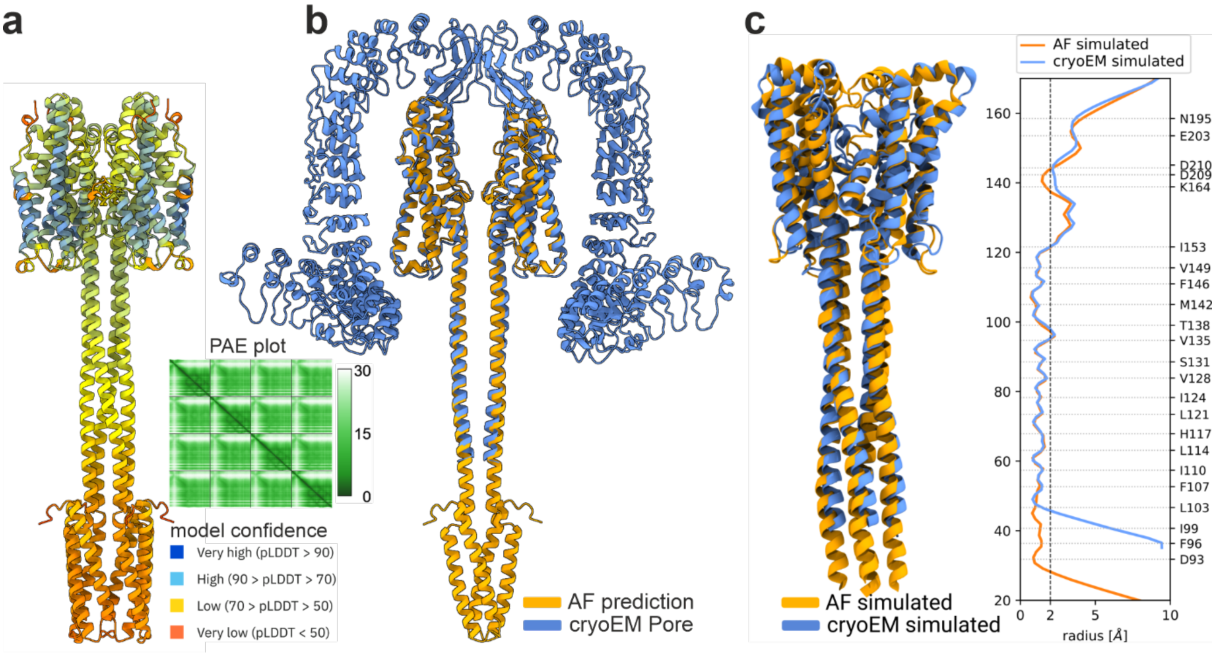

Supplementary figure 8: Comparison of AlphaFold2 prediction of N-terminal α-LTX central core with the cryo-EM structure of the α-LTX pore. a AlphaFold2 prediction of a tetrameric N-terminal central core of α-LTX (CD-HBD; residues E21-E360) colored by model confidence (pLDDT). The predicted error estimates (PAE) for the prediction are shown in shades of green. **b** Overlay of the AlphaFold2 prediction (orange) with the cryo-EM structure of the α-LTX pore (blue). The RMSD between 221 pruned atom pairs is 0.91 Å. Shown are two opposing subunits. **c** Results of MD simulations of 1 µs, starting from the AlphaFold2 (orange, residues C91 to Y260) and the cryo-EM structure (blue, residues N105 to Y260), respectively. Left: Overlay of both resulting structures. Right: Comparison of the average radius profiles of these structures. One can see the excellent agreement, supporting the close similarity of AlphaFold2 and cryo-EM structures.

Supplementary figure 9

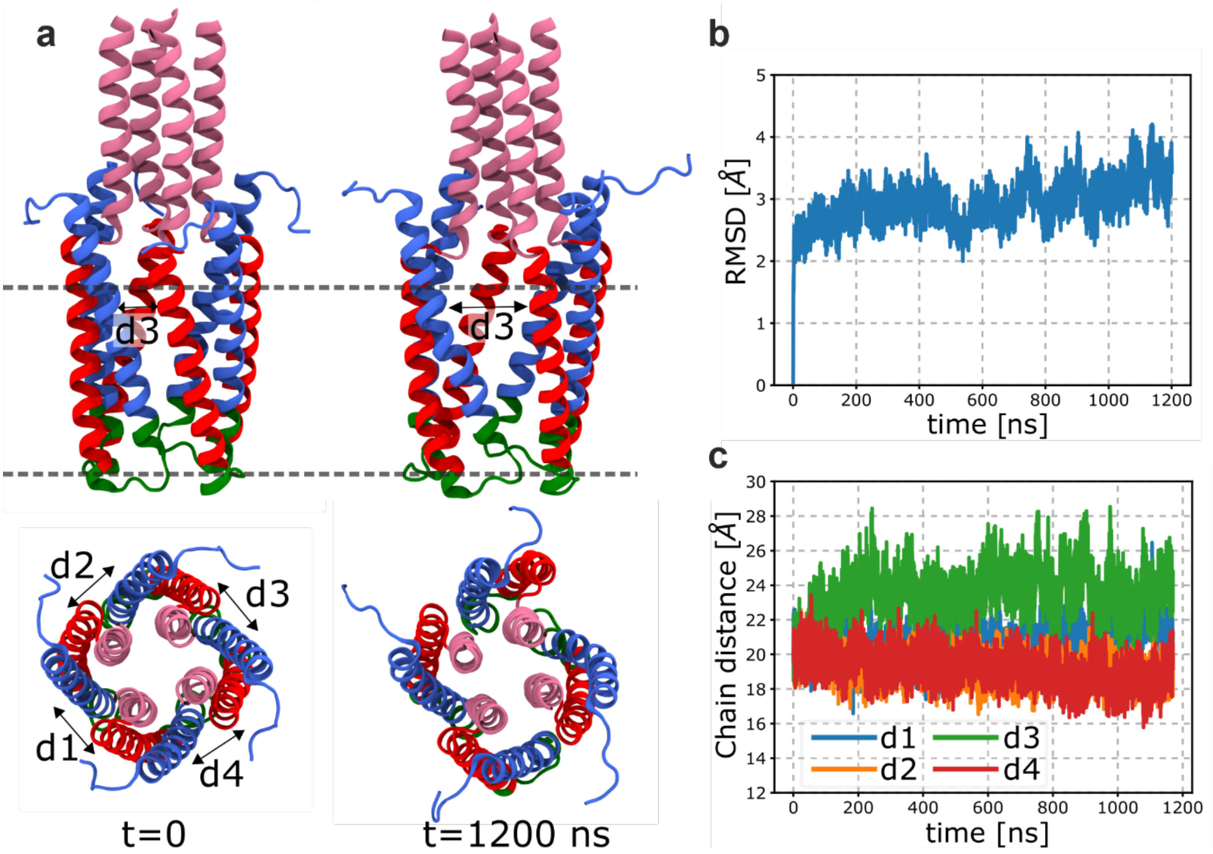

Supplementary figure 9: MD-simulation of the conformational dynamics and stability of the α-LTX transmembrane pore inside the membrane. a Structural changes in the TMD of α-LTX predicted by AlphaFold2 inside the membrane during 1200 ns simulation time, obtained via MD simulations (0.15 M $CaCl_2$, no electric field). Chosen are a side and a top view in cartoon representation (lipids not shown). The different orientations help to visualize the residual fluctuations of the TMD. **b,c** RMSD of the protein backbone atoms (**b**) and the average distance between the helix α1 (residues T26-L57) from one subunit to helix α3 (residues G61-G90) of the neighboring subunit (**c**) are shown versus the simulation time in the system with 0.15 M $CaCl_2$. Initially, one of the four distances (d3) increases slightly. Importantly, after about 100 ns, none of the distances increases any more. Thus, the simulations suggest that the overall TMD is stable.

Supplementary figure 10

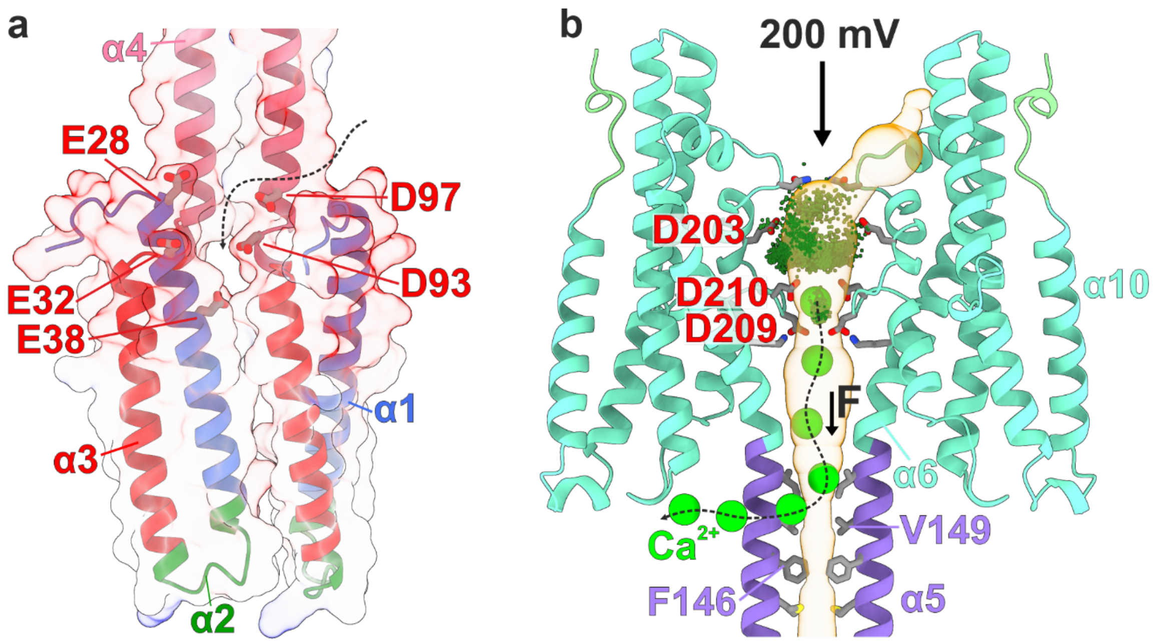

Supplementary figure 10: Potential cation gates of α-LTX. a The lateral cation entry site between the distal end of the coiled-coil and the transmembrane domain. Negatively charged residues lining the gate are indicated. The respective MD simulations are shown in Figure 3. **b** The HBD (cyan) and the proximal end of the tetrameric coil (purple) form a continuous channel. The HBD loops form a central cavity with three layers of channel-lining negatively charged residues (E203; D209; D210). MD simulations show that Ca^{2+} ions enter this cavity to bind strongly to the site formed between the second and third layers (D209; D210; diameter 4Å; Figure 3), but do not further enter the coiled-coil stalk. The majority of Ca^{2+} enters the upper opening of the channel, but is less localized, approximately at the level of the first layer (E203; diameter 6Å; Figure 3). The small green dots show the superposition of Ca^{2+} ions from all 1000 frames (step 1 ns) of an MD simulation with an applied electrical potential difference of 200 mV. When an additional pulling force is applied (see methods), Ca^{2+} ions do pass through the strong binding site but then they exit laterally and do not translocate through the stalk. The large green spheres show representative frames of such a translocation event.

Supplementary figure 11

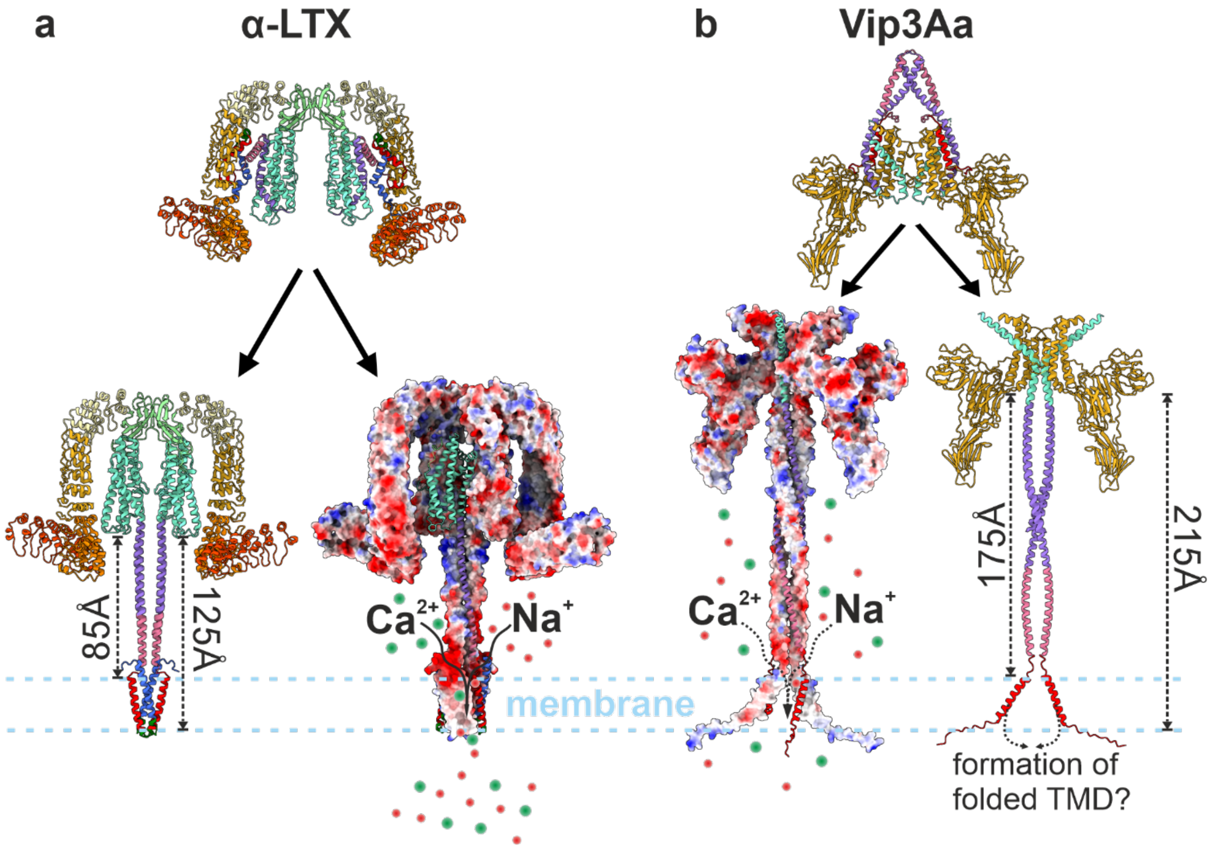

Supplementary figure 11: Comparison of the pore-forming toxins α-LTX (a) and Vip3A[2] (b). Both toxins form channels which are highly cation selective. For each toxin, the prepore state is shown on top and the pore state (upon membrane insertion) at the bottom. Note the characteristic umbrella-like shape of both toxins and also the central tetrameric coiled-coil stalk, required for membrane insertion. The tetrameric assembly of the N-terminus including the potential transmembrane domains was predicted by Alphafold2 in both cases. Surface electrostatics depict the amphipathic properties of the transmembrane domains (TMDs). It should be noted that the prediction of the Vip3A TMD was less stable than for the TMD of α-LTX, and its properties will require further analysis. In contrast to α-LTX, Vip3A does not contain a stabilizing disulfide in this region, suggesting that such an arrangement might contain only one transmembrane helix per monomer and not two like in α-LTX. The cation translocation pathway in Vip3A remains unclear, as does whether the lower end of the stalk provides a lateral entry gate for cations, similar to α-LTX. Further studies are needed to unravel such similarities in the channel-forming mechanisms of these until recently unrelated cation channel-forming toxins.

Supplementary figures 12:

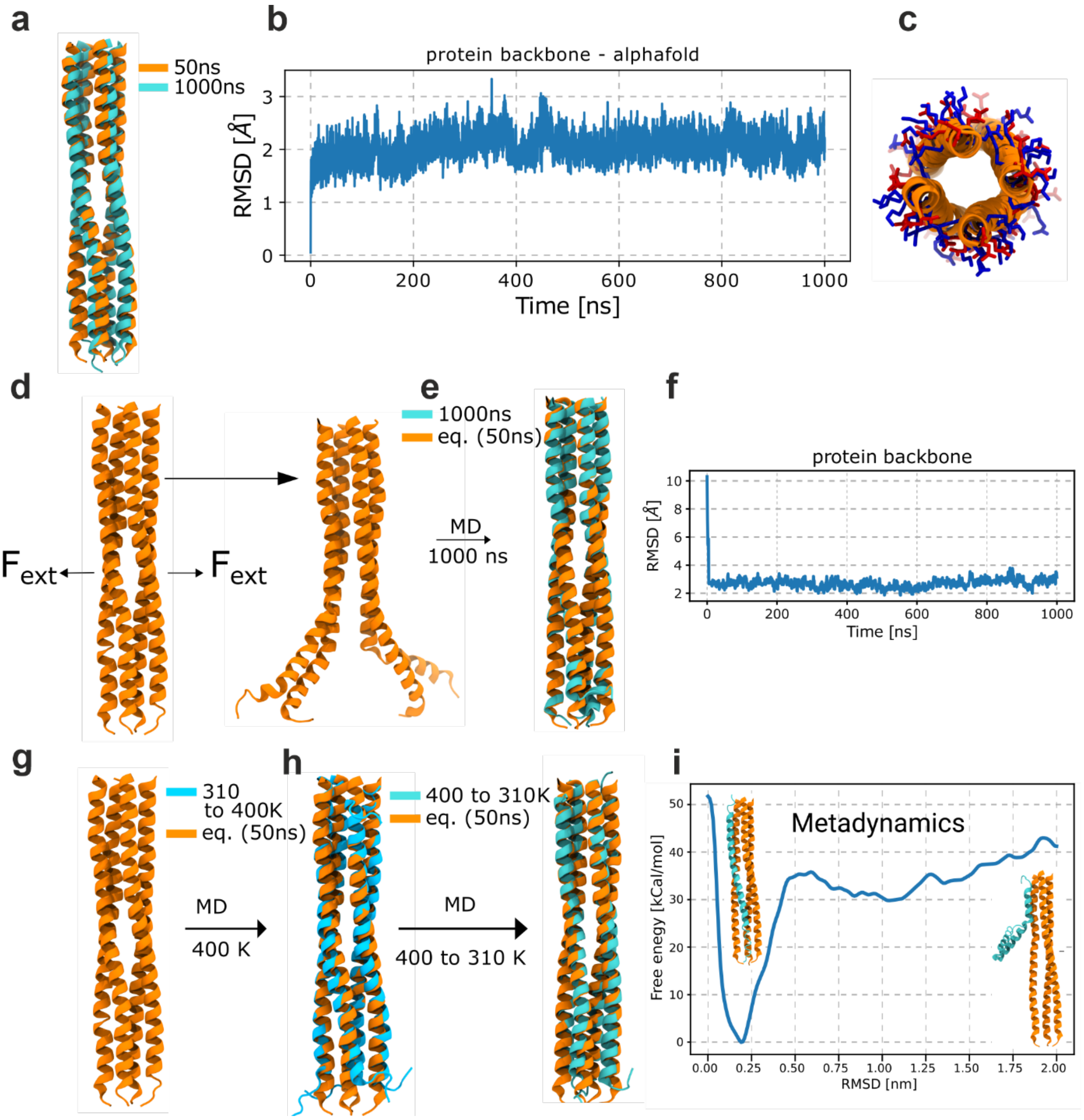

Supplementary figure 12: MD simulations of the stalk as predicted by AlphaFold2 to probe its stability. a Overlay of the two protein structures from the MD simulations of the stalk (residues C91-D155) extracted from the 50 ns (orange) and 1000 ns (cyan) of the simulation, showing the stalk stability. **b** The RMSD of the stalk backbone atoms also showing high stability. **c** Top view of the stalk together with the negatively (red) and positively (blue) charged residues in sticks representation. The specific arrangement of the polar residues outside and the hydrophobic residues inside the pore contribute to the high stability of the stalk. **d** Steered MD simulations of the AlphaFold2 structure with an external force, applied to the center of mass of the four chains towards outside of the pore. **e** A structure from the steered MD was adopted and a normal MD was performed for 1 µs. The overlay of the structure at the end of this simulation and the equilibrated structure from the normal MD in **a** is represented, showing that the distorted structure approaches the initial structure. **f** RMSD of the protein backbone atoms from the simulations in **e**, showing that the

distorted structure abruptly approaches the initial structure and remains stable. **g** Simulation results of the equilibrated stalk (50 ns) at 400 K, distorting the structure. The overlay of the structure at the end of the simulation (blue) with the equilibrated structure (orange) is shown. **h** Starting with the structure at the end of the simulation at 400 K, the temperature was gradually decreased to 310 K. The overlay of the end structure (cyan) and the equilibrated structure (orange) is shown in cartoon representation. Both results in **e** and **h** indicate that the stalk structure is close to a deep minimum of the system free energy. **i** Free energy of stalk formation, starting from a combination of three monomers from the tetrameric coiled-coil of the pore state (residues C91-D155, orange cartoon), with one monomer of the prepore structure (cyan), as shown at the right side. The structure at its minimum (at ~0.19 nm) is shown at the left side.

Supplementary figure 13:

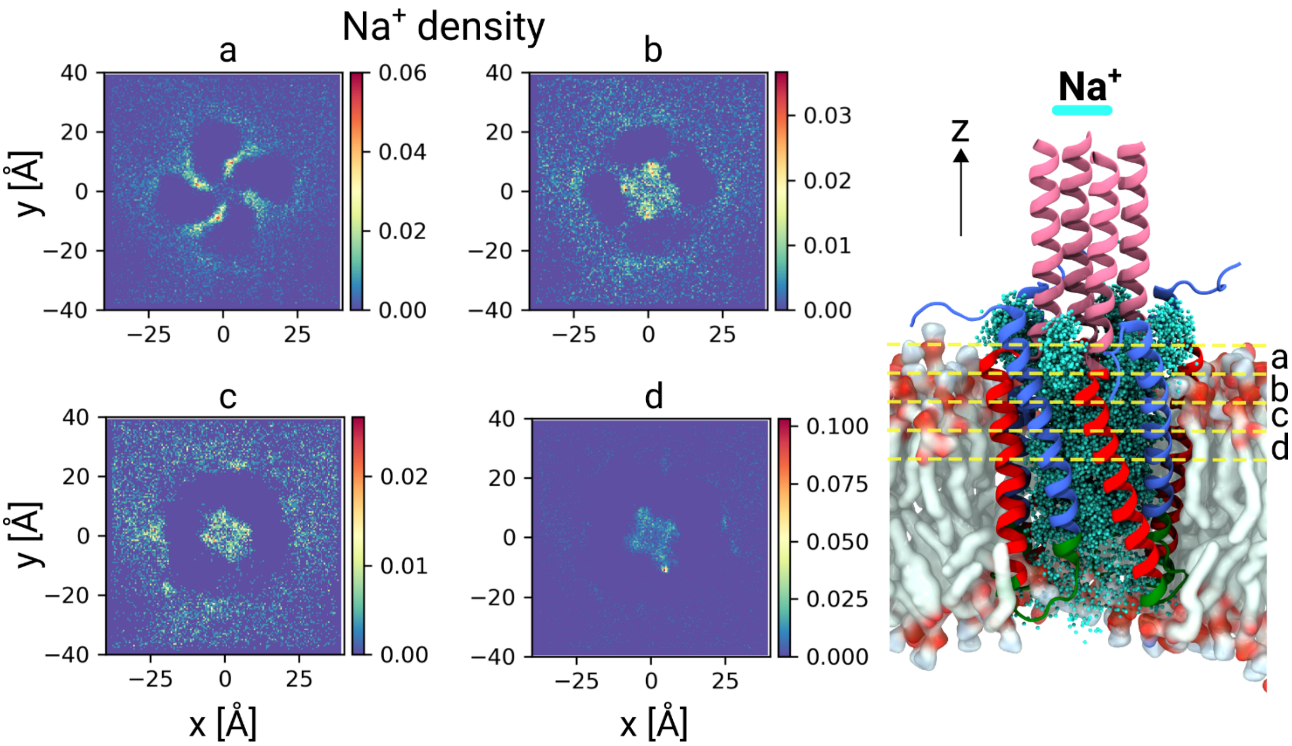

Supplementary figure 13: Simulations of the membrane part with Na^{+}. Density maps of Na^{+} ions from the MD simulation of the TMD of the AlphaFold2 prediction of α-LTX for different cross sections of the protein along the z-axis and width of 5 Å. The corresponding density maps for Ca^{2+} are shown in Figure 3d.

Supplementary figure 14

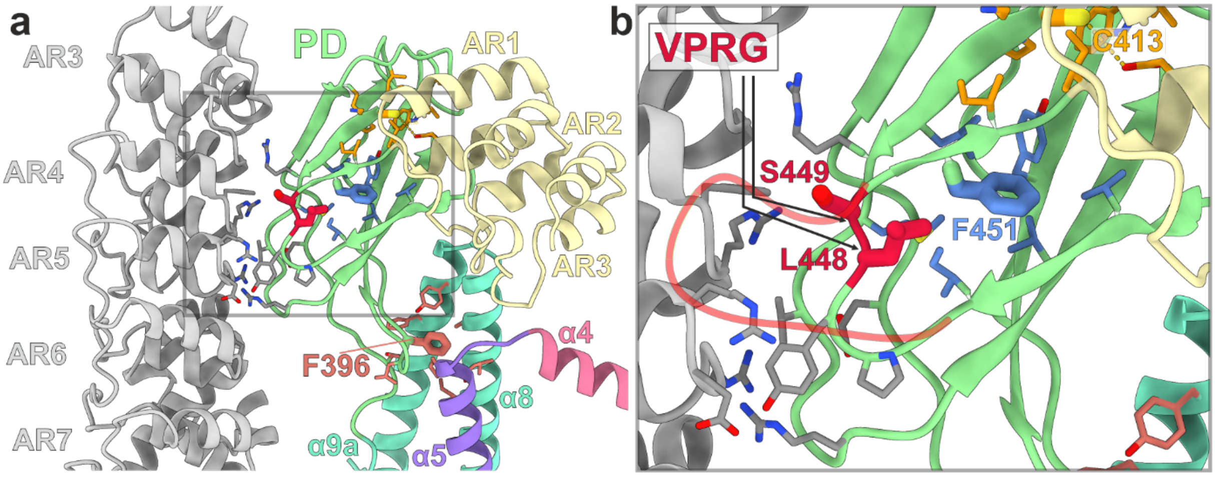

Supplementary figure 14: Effect of the mutant α-LTX^{N4C}[2]. **a** Region of inter- and intrasubunit interactions between the PD and the ARD. For clarity, three hydrophobic pockets with the central atoms C413, F451 and F396 are highlighted in orange, blue and pink, respectively. Residues L448 and S449, between which the VPRG sequence is inserted in the α-LTX^{N4C} mutant[2], are colored in red. **b** A loop created by the VPRG insertion (pale red line) was roughly modeled in coot and superimposed on the WT model to highlight potential clashes with the ARD (gray) of the neighboring subunit, explaining the abolished ability of the α-LTX^{N4C} variant to form tetramers.

Supplementary figure 15

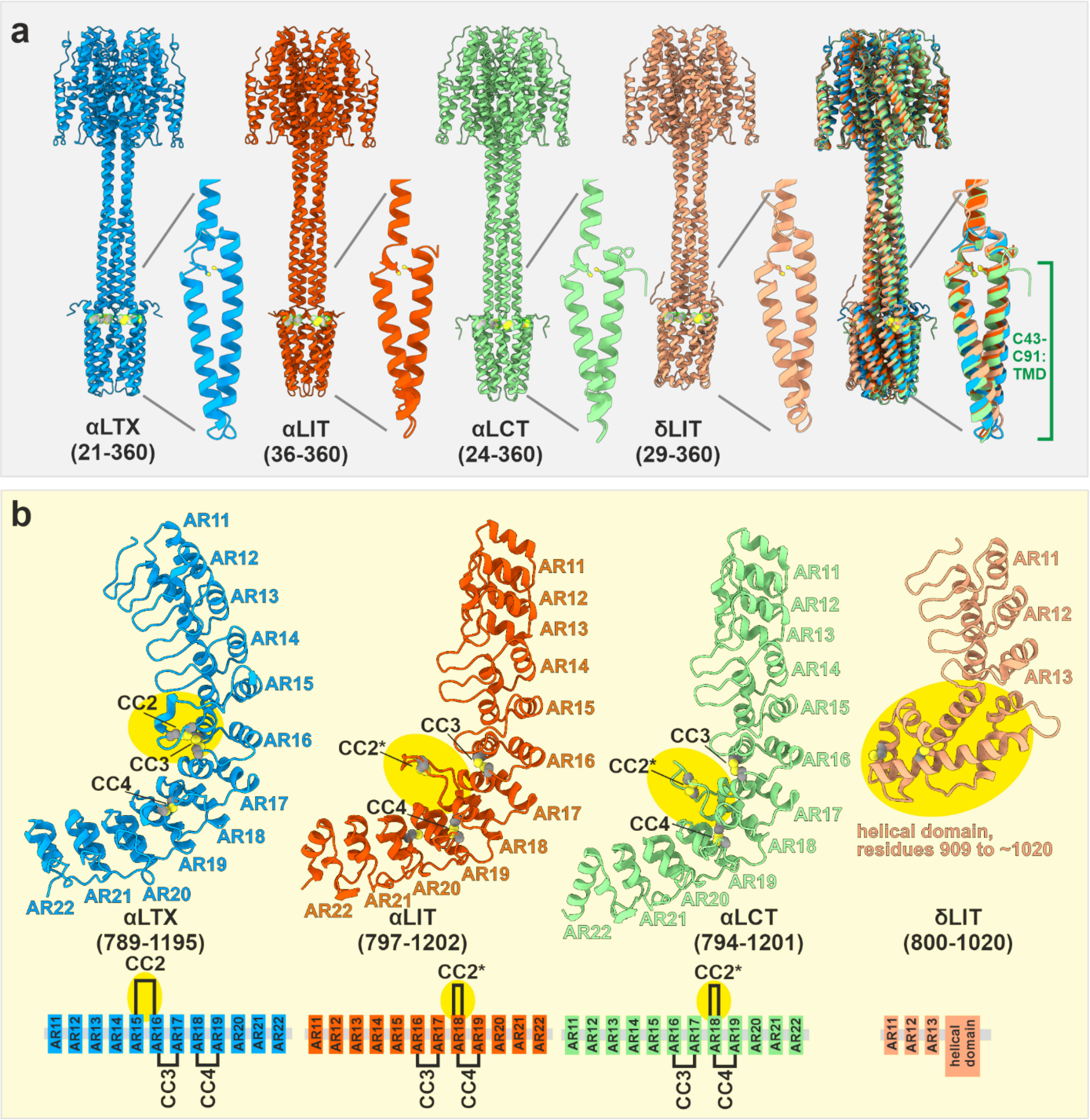

Supplementary figure 15: Comparison of AlphaFold2 predictions for different latrotoxins (α-LTX (vertebrate-specific), α-LIT, δ-LIT (insect-specific) and α-LCT (crustacean-specific)). a AlphaFold2 predictions of tetramers of the tetrameric central-core (CD; HBD) of different LTXs. The motif of an N-terminal TMD stabilized by a conserved disulfide bridge (yellow) is predicted in all analyzed latrotoxins. All latrotoxins present a remarkable similarity to α-LTX in this region, with RMSDs of 1.01 Å (α-LIT, 290 pruned atom pairs), 1.08 Å (α-LCT, 243 pruned atom pairs) and 1.03 Å (δ-LIT, 280 pruned atom pairs) **b** AlphaFold2 predictions of monomers of the C-terminal part (ARD) for different latrotoxins. An elongated loop containing a stabilizing disulfide bridge is present between AR15 and AR16 in α-LTX and within AR18 in α-LIT and α-LCT. In contrast, δ-LIT is truncated and ends with a small and highly negatively charged helical domain that contains two cysteines.

**Data collection**			
Microscope	Titan Krios G4 (Selectris X, E-CFEG)		
Voltage (kV)	300		
Camera	Falcon 4i		
Pixel size (Å)	0.58		
Tilt angle	Micrographs	Initial particles	Particles prepore / pore in final stack
0°	8,417	447,887	124,318 / 13,141
21°	23,158	504,019	96,514 / 12,619
30°	14,167	425,266	33,497 / 17,164
32°	8,755	234,017	35,444 / 6,372
35°	1,137	33,715	12,846 / 1,347
40°	965	20,343	7,324 / 772
42°	11,926	320,948	59,114 / 8,392
42°	2,005	74,390	19,367 / 3,170
50°	4,700	359,204	52,821 / 7,733
60°	738	16,354	860 / 261
total number of particles	2,436,143		
Number of frames	918-1225		
Number of fractions	49-51		
Total electron dose ($e^-/Å^2$)	50		
Defocus range (µm)	-0.3 – -1.7		
**Atomic model composition**	**Prepore**	**Pore**	
Chains	4	4	
Symmetry imposed	C1	C1	
Non-hydrogen (protein) atoms	36,980	31,628	
Residues	4,700	4320	
particle substack	442,105	70,971	
Ligand atoms	-	-	
**Refinement (Phenix)**			
RMSD bond	0.002	0.004	
RMSD angle	0.571	0.709	
Model to map fit, CC mask	0.72 (0.73)	0.61 (0.60)	
Model to map fit, CC box	0.57 (0.86)	0.67 (0.77)	
Resolution (FSC@0.143, Å)	2.71 (3.15)	3.61 (3.69)	
B-factor (mean, $Å^2$)	116.04	89.73	
**Validation**			
Clashscore	8.75	9.71	
Ramachandran outliers (%)	0.02	0.02	
Ramachandran favoured (%)	96.12	96.29	
Molprobity score	1.73	1.76	
EMRinger score	1.46 (1.18)	1.40 (0.84)	

Supplementary table 1: Cryo-EM data collection and refinement statistics of α-LTX. The refinement in Phenix was done against hybrid maps obtained by Phenix.combine_focussed_maps after multi-body refinement (Supplementary figure 2). The presented refinement statistics are for the hybrid maps (resolution is the reported resolution from multi-body refinement), and in brackets for the same model against the original, not locally refined nor combined maps after 3D-refinement and sharpening in RELION4.

$Na^+/Ca^{2+}/La^{3+}$	Na^+	Na^+ with EF	Ca^{2+}	Ca^{2+} with EF	La^{3+}	La^{3+} with EF
ext. to int.	51	136	6	61	0	0
int. to ext.	43	19	4	1	0	0

Supplementary table 2: Number of TMD permeation events. Number of permeation events of the cations across the protein membrane part during the production simulation of 1200 ns, starting from the AlphaFold2 structure, for different ions and with and without the applied electric field corresponding to an electrical potential difference of 100 mV. As expected, without an electric field the number of transitions is, within statistical uncertainties, identical in both directions. Application of an electric field induces a directional bias due to the additional gradient in potential energy which is particularly pronounced for the divalent cation.

$Na^+/Ca^{2+}/La^{3+}$	Na^+	Na^+ with EF	Ca^{2+}	Ca^{2+} with EF	La^{3+}	La^{3+} with EF
ext. to int.	25	19	170	76	-	-
int. to ext.	31	18	251	93	-	-

Supplementary table 3. Average time duration of TMD permeation events. Average time duration (in ns) of the permeation events of cations across the membrane part, as listed in Supplementary Table2. As expected from transition state theory, despite application of an electric field the transition times remain similar in both directions but become shorter. The transition times for the divalent cation is approx. 3 times slower as compared to the monovalent cation.

Prepore	Inter-molecular interfaces		
**Fragment A residues**	**Fragment B residues**	**Δ^{i}G (A-B) [kcal/mol]**	**Interface area (A-B) [Å^2]**
I454-G1195	Q351-D453	-3.3 ± 0.4	692.4 ± 12.9
A156-K350	E21-C91	-0.7 ± 0.6	30.9 ± 22.2
**Prepore**	**Intra-molecular interfaces**		
**Fragment A residues**	**Fragment B residues**	**Δ^{i}G (A-B) [kcal/mol]**	**Interface area (A-B) [Å^2]**
A156-K350	S92-D155	-21.4 ± 1.7	1595.0 ± 46.2
I454-G1195	E21-C91	-25.0 ± 1.4	1447.3 ± 59.5
S92-D155	E21-C91	-15.8 ± 0.4	735.7 ± 9.3
I454-G1195	S92-D155	-3.7 ± 0.7	310.1 ± 5.1
Q351-D453	S92-D155	-5.2 ± 0.1	204.5 ± 2.2

Pore	Inter-molecular interfaces		
**Fragment A residues**	**Fragment B residues**	**Δ^{i}G (A-B) [kcal/mol]**	**Interface area (A-B) [Å^2]**
S92-D155	S92-D155	-21.5 ± 0.8	1297.2 ± 26.0
S92-D155 *(diagonal contact)*	S92-D155 *(diagonal contact)*	-5.10 ± 0.2	166.8 ± 3.0
A156-K350	A156-K350	-4.1 ± 0.5	856.9 ± 35.7
I454-G1195	Q351-D453	-2.2 ± 0.8	714.3 ± 21.5
E21-C91	E21-C91	-4.7 ± 1.8	429.4 ± 72.1
A156-K350	S92-D155	-0.7 ± 0.04	23.4 ± 1.6

Supplementary table 4: Interfaces between α-LTX subunits in the tetrameric assembly. Each of the four α-LTX monomers were segmented into five fragments, and mutual interactions between fragments A and B as indicated were analyzed by the Pisa server[3]. Residues E21-C91 correspond to helices α1-α3 that form the transmembrane domain in the pore, residues S92-D155 form the tetrameric coiled-coil stalk in the pore state, residues A156-K350 corresponds to the HBD excluding helix α5 which is part of the stalk in the pore state, residues Q351-D453 correspond to the PD, and I454-G1195 to the ARD. Only interactions with significant relevance for stability are shown as mean values of clockwise intermolecular interactions within the tetramer +/- SD. Only within the stalk region, interactions of diagonally opposing monomers are also contributing significantly to complex stability. Interactions between residues E21-D155, which form the stalk and transmembrane domain in the pore state, contribute by far strongest for the stability of the tetrameric α-LTX pore. Since intermolecular interactions of these residues are missing in the prepore state, the tetrameric prepore is much weaker than the pore assembly. However, in the prepore state, residues E21-D155 are involved in intramolecular interactions which sum up to slightly higher solvation free energies (Δ^{i}G) than their intermolecular interactions in the pore state, indicating that despite the high solvation free energy of the formed coiled-coil stalk, the prepore to pore transition is not an exothermic process.

Supplementary References

1. Tan, Y. Z. *et al.* Addressing preferred specimen orientation in single-particle cryo-EM through tilting. *Nature methods* **14,** 793–796; 10.1038/nmeth.4347 (2017).
2. Núñez-Ramírez, R. *et al.* Molecular architecture and activation of the insecticidal protein Vip3Aa from Bacillus thuringiensis. *Nature communications* **11,** 3974; 10.1038/s41467-020-17758-5 (2020).
3. Krissinel, E. & Henrick, K. Inference of macromolecular assemblies from crystalline state. *Journal of molecular biology* **372,** 774–797; 10.1016/j.jmb.2007.05.022 (2007).
