# Supplementary material for: Structural basis of α-latrotoxin transition to a cation-selective pore
Source: Nat Commun. 2024 Oct 3;15:8551. doi: 10.1038/s41467-024-52635-5 (PMC11449929; doi:10.1038/s41467-024-52635-5)
Supplement: Supplementary file 3 — Description of Additional Supplementary Files [file 41467_2024_52635_MOESM3_ESM.pdf]

Description of Additional Supplementary Files:

Supplementary Movie 1: Cryo-EM map of α-LTX prepore and the corresponding molecular model.

Supplementary Movie 2: Cryo-EM map of α-LTX pore and the corresponding molecular model.

Supplementary Movie 3: Conformational variability of the prepore state of α-LTX. Shown are morphs between cryo-EM 3D classes of the α-LTX prepore state (Supplementary figure 2).

Supplementary Movie 4: Time evolution of the TMD region during 1200 ns, as obtained from the MD simulations. The initial and final configurations of this video are shown in Supplementary figure 9.

Supplementary Movie 5: Simulations of the TMD with Na+ and Ca2+ ions for an applied electric field corresponding to an electric potential difference of 100 mV. Shown are the protein and the different ions for a time duration of approx. 150 ns. The time evolution is identical for both ions, highlighting the faster translocation of Na+ through the channel. The lipids are hidden for better clarity.

Supplementary Movie 6: Ca2+ -binding stabilizes the tetrameric prepore in a narrow conformation and thereby assists pore formation. Morphs between different cryo-EM 3D classes of the α-LTX prepore and pore state. Note the "breathing motions" of the central channel in the prepore state. Narrowing of the channel and its stabilization in this conformation by Ca2+ assists the pore formation events.

Supplementary Movie 7: The video shows a simplified model of α-LTX prepore→pore transition, membrane penetration, channel formation and Ca2+ translocation obtained after morphing between the structures in the prepore and the pore state. It should be noted that structures of possible intermediate states, as shown in this animation, are not yet available.

Supplementary Movie 8: α-LTX prepore→pore transition (for description see Supplementary movie 7), as seen from the bottom- and top-view.
