# Supplementary material for: Structural basis of α-latrotoxin transition to a cation-selective pore
Source: Nat Commun. 2024 Oct 3;15:8551. doi: 10.1038/s41467-024-52635-5 (PMC11449929; doi:10.1038/s41467-024-52635-5)
Supplement: Supplementary file 12 — Reporting Summary [file 41467_2024_52635_MOESM12_ESM.pdf]

Corresponding author(s): Christos Gatsogiannis, Andreas Heuer

Last updated by author(s): Aug 15, 2024

Reporting Summary

Nature Portfolio wishes to improve the reproducibility of the work that we publish. This form provides structure for consistency and transparency in reporting. For further information on Nature Portfolio policies, see our Editorial Policies and the Editorial Policy Checklist.

Statistics

For all statistical analyses, confirm that the following items are present in the figure legend, table legend, main text, or Methods section.

n/a	Confirmed	
☐	☒	The exact sample size (*n*) for each experimental group/condition, given as a discrete number and unit of measurement
☒	☐	A statement on whether measurements were taken from distinct samples or whether the same sample was measured repeatedly
☒	☐	The statistical test(s) used AND whether they are one- or two-sided<br>*Only common tests should be described solely by name; describe more complex techniques in the Methods section.*
☒	☐	A description of all covariates tested
☒	☐	A description of any assumptions or corrections, such as tests of normality and adjustment for multiple comparisons
☐	☒	A full description of the statistical parameters including central tendency (e.g. means) or other basic estimates (e.g. regression coefficient) AND variation (e.g. standard deviation) or associated estimates of uncertainty (e.g. confidence intervals)
☒	☐	For null hypothesis testing, the test statistic (e.g. *F*, *t*, *r*) with confidence intervals, effect sizes, degrees of freedom and *P* value noted<br>*Give P values as exact values whenever suitable.*
☒	☐	For Bayesian analysis, information on the choice of priors and Markov chain Monte Carlo settings
☒	☐	For hierarchical and complex designs, identification of the appropriate level for tests and full reporting of outcomes
☒	☐	Estimates of effect sizes (e.g. Cohen's *d*, Pearson's *r*), indicating how they were calculated

Our web collection on statistics for biologists contains articles on many of the points above.

Software and code

Policy information about availability of computer code

Data collection	EPU version 3.2.0.4776 ; Custom code used to generate data described in the manuscript is available in the source data file published alongside this manuscript.
Data analysis	RELION ver.4.01, CTFFIND4 ver.4.1.14, crYOLO ver. 1.8.3-1.9.1, PHENIX ver.1.20.1, WinCOOT ver.0.9.8.1, UCSF ChimeraX ver.1.7.dev2023, Alphafold 2 ver. 2.3.1, Corel Draw 2020, angdist ver.1.2, MD SIMULATION (GROMACS ver. 2019.6, plumed ver. 2.6.4), MD ANALYSIS (ver. 2.3.0), ONLINE SERVERS (PISA, 3D-FSC, moleonline), CHARMM-GUI, HOLE (v.2.2.004), VMD(1.9.3).

For manuscripts utilizing custom algorithms or software that are central to the research but not yet described in published literature, software must be made available to editors and reviewers. We strongly encourage code deposition in a community repository (e.g. GitHub). See the Nature Portfolio guidelines for submitting code & software for further information.

Data

Policy information about availability of data

All manuscripts must include a data availability statement. This statement should provide the following information, where applicable:

- Accession codes, unique identifiers, or web links for publicly available datasets
- A description of any restrictions on data availability
- For clinical datasets or third party data, please ensure that the statement adheres to our policy

Cryo-EM maps were deposited in the Electron Microscopy Data Bank (EMDB ID: EMD-51494, EMD-51465 EMD-51467, EMD-51468, EMD-51469, EMD-51472,

EMD-51473, EMD-51474, EMD-51475, EMD-51492, EMD-51495, EMD-51476, EMD-51479, EMD-51485, EMD-51484, EMD-51488, EMD-51490). Coordinates were deposited in the Protein Data Bank (PDB ID 9GO9, 9GOA) and are available upon publication. The raw cryo-electron movies and particle coordinates have been deposited to EMPIAR (EMPIAR-XXX) and are available upon publication. The MD input files, starting structures and final structures as representative configurations can be found under https://zenodo.org/records/12663805.

Research involving human participants, their data, or biological material

Policy information about studies with human participants or human data. See also policy information about sex, gender (identity/presentation), and sexual orientation and race, ethnicity and racism.

Reporting on sex and gender	N/A
Reporting on race, ethnicity, or other socially relevant groupings	N/A
Population characteristics	N/A
Recruitment	N/A
Ethics oversight	N/A

Note that full information on the approval of the study protocol must also be provided in the manuscript.

Field-specific reporting

Please select the one below that is the best fit for your research. If you are not sure, read the appropriate sections before making your selection.

☒ Life sciences ☐ Behavioural & social sciences ☐ Ecological, evolutionary & environmental sciences

For a reference copy of the document with all sections, see nature.com/documents/nr-reporting-summary-flat.pdf

Life sciences study design

All studies must disclose on these points even when the disclosure is negative.

Sample size	90,215 cryo-EM movies were collected using the EPU software, out of which 75,968 were selected for further processing based on local resolution limit and estimated defocus. Further details are listed in supplementray table 1. The sample size for cryo-EM data was chosen large enough to allow high resolution reconstructions even from rare particle subpopulations representing alternative conformations like the Pore state of latrotoxin. After particle sorting, the final prepore particle stack contained particles from 49,434 of the original movies. This dataset was sufficient for high-resolution cryo-EM image analysis as shown throughout the manuscript. For MD simulations, the simulation box contains 90,000 - 200,000 atoms, depending on the size of the analysed protein. The sample size was chosen such that the protein did not display any short-range interaction with itself via periodic boundary conditions.
Data exclusions	Particles that did not present a tetrameric oligomerisation state were excluded by manual inspection of 2D- or 3D-sorted particle classes. Data with high heterogeneity was isolated into subclasses by 3D classification, of which the lower-resolved ones were only used to visualize the conformational heterogeneity as presented in Supplementary movies 3 and 6. Data of low quality were excluded during single particle image processing in order to reach high-resolution using statistical methods. These exclusion criteria, as implemented in Relion, are well established, and are a common practice in the cryo-EM field.
Replication	All cryo-EM experiments presented in this manuscript were obtained from a single sample of latrotoxin. The results are in excellent agreement, but of higher quality, than previously performed experiments on other latrotoxin samples from which we collected and analyzed cryo-EM data before (data not shown, those experiments reached ~4.0 Angstroem resolution). For EM experiments, several negative stain EM and cryo-EM grids were prepared. We selected several of the highest quality grids for data acquisition. Replication of MD simulations: for each ion concentration of different parts of the pore structure one sample was simulated. We confirm that all attempts to replicate our experimental findings were successful.
Randomization	Randomization was performed by splitting particle stacks randomly into half-sets and calculating the corresponding resolution estimates from the Fourier shell correlation of derived half-maps at the 0.143 threshold criterium. For MD simulations, at the beginning of the simulations, the atomic positions and velocities are randomized to represent an initial state that resembles the system at a given temperature and then to achieve equilibration.
Blinding	Blinding is not relevant to this structural study as it does not contain experiments with groups of individuals such as in medical field studies. Particle assignment to half-sets and the corresponding resolution estimation were performed automatically by processing software packages. For MD simulations, blinding is not relevant.

Reporting for specific materials, systems and methods

We require information from authors about some types of materials, experimental systems and methods used in many studies. Here, indicate whether each material, system or method listed is relevant to your study. If you are not sure if a list item applies to your research, read the appropriate section before selecting a response.

Materials & experimental systems

n/a	Involved in the study
☒	☐ Antibodies
☒	☐ Eukaryotic cell lines
☒	☐ Palaeontology and archaeology
☒	☐ Animals and other organisms
☒	☐ Clinical data
☒	☐ Dual use research of concern
☒	☐ Plants

Methods

n/a	Involved in the study
☒	☐ ChIP-seq
☒	☐ Flow cytometry
☒	☐ MRI-based neuroimaging

Plants

Seed stocks	*Report on the source of all seed stocks or other plant material used. If applicable, state the seed stock centre and catalogue number. If plant specimens were collected from the field, describe the collection location, date and sampling procedures.*
Novel plant genotypes	*Describe the methods by which all novel plant genotypes were produced. This includes those generated by transgenic approaches, gene editing, chemical/radiation-based mutagenesis and hybridization. For transgenic lines, describe the transformation method, the number of independent lines analyzed and the generation upon which experiments were performed. For gene-edited lines, describe the editor used, the endogenous sequence targeted for editing, the targeting guide RNA sequence (if applicable) and how the editor was applied.*
Authentication	*Describe any authentication procedures for each seed stock used or novel genotype generated. Describe any experiments used to assess the effect of a mutation and, where applicable, how potential secondary effects (e.g. second site T-DNA insertions, mosiacism, off-target gene editing) were examined.*
